# Supplementary material for: Minimal‐Invasive 3D Laser Printing of Microimplants in Organismo
Source: Adv Sci (Weinh). 2024 Jun 12;11(30):2401110. doi: 10.1002/advs.202401110 (PMC11321634; doi:10.1002/advs.202401110)
Supplement: Supplementary file 1 — Supporting Information [file ADVS-11-2401110-s002.docx]

# **Supplemental Information**

**Fig. S1: UV cured spheres explanted from *Drosophila* embryos after timelapse microscopy.**

**
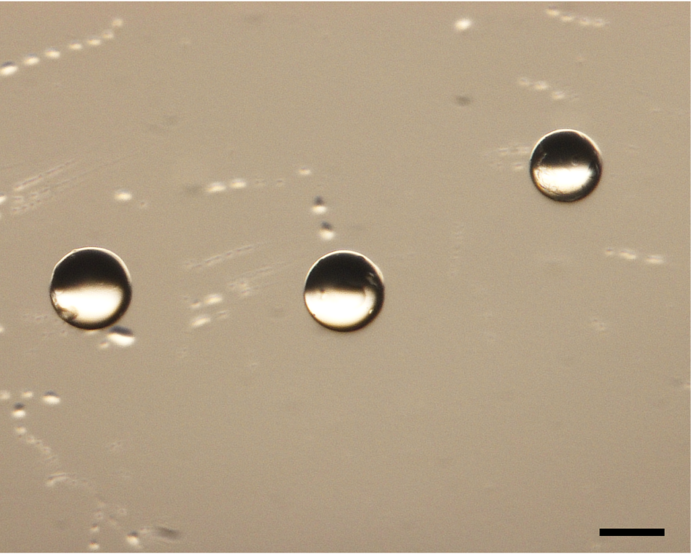
**

Scale bar: 100 µm.

**Fig. S2: Total distance traveled of uncured droplets and UV cured spheres in *Drosophila* embryos before cellularization.**

**
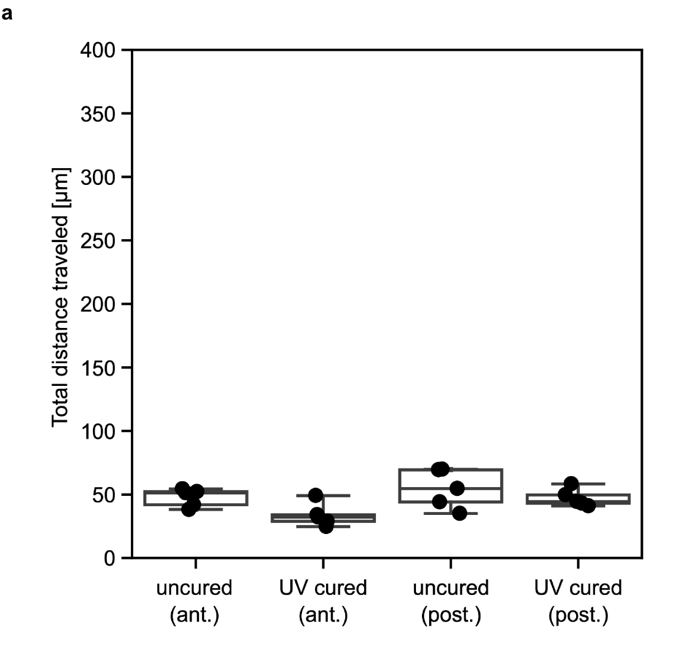
**

**a** Quantification of total distance traveled of the uncured and UV cured, microinjected IP-PDMS deposits at the anterior (ant.) and posterior (post.) poles of the *Drosophila* embryos. Spheres were tracked in 10 min increments in timelapse microscopy images from the last mitotic wave until end of cellularization. Boxplots include data from 5 embryos each with boxes indicating 25-75% percentiles and whiskers 10-90% percentiles. Individual data points shown as dots.

**Fig. S3: *In vivo* one-photon photopolymerized IP-PDMS spheres allow local re-shaping of the *Drosophila* cephalic furrow.**

**
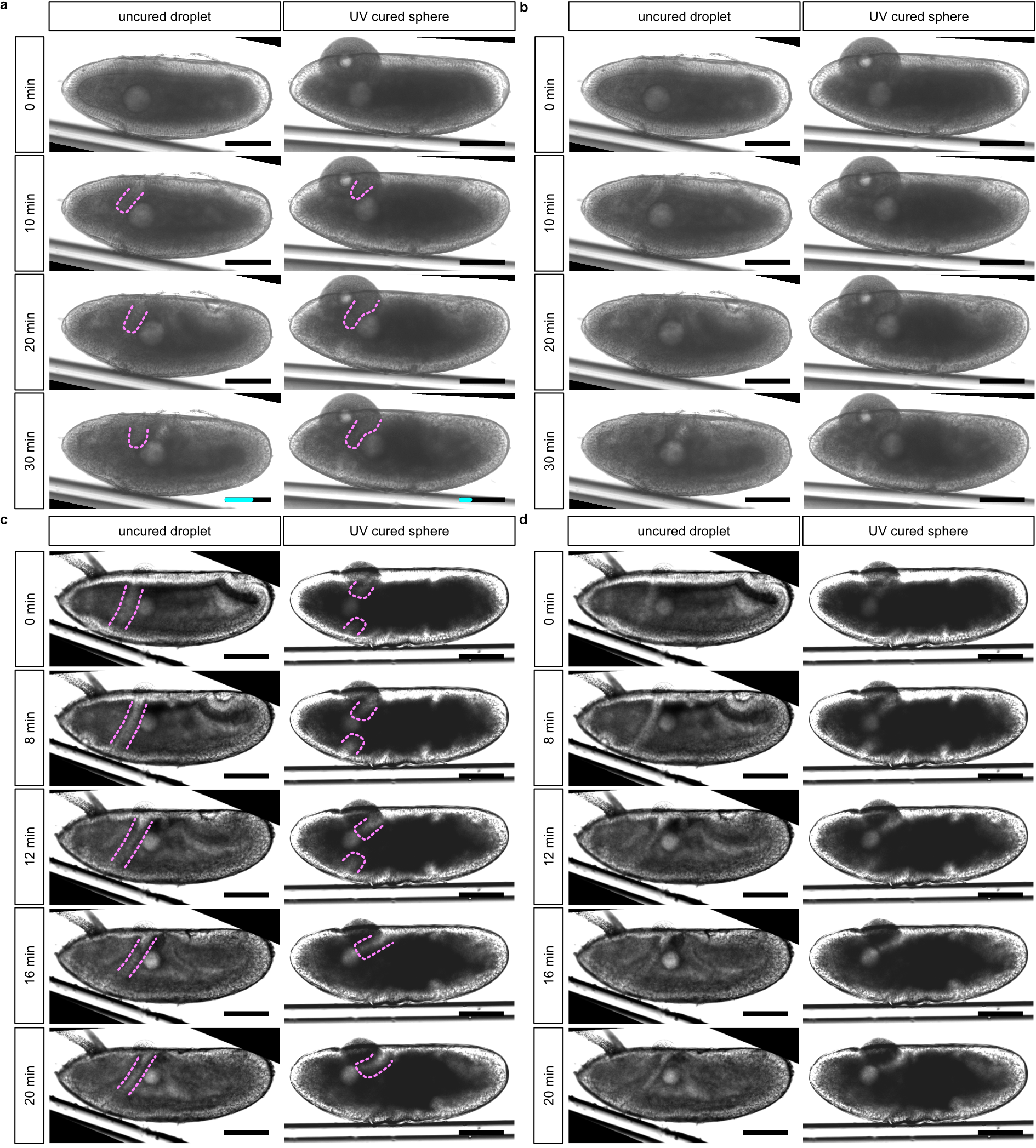
**

**a/b** Representative timelapse transmission microscopy of early *Drosophila* embryos during cephalic furrow formation microinjected with IP-PDMS with (a) and without (b) overlays. Microinjected IP-PDMS deposits were either left uncured (left panels) or cured by UV light exposure (right panels). Cyan scale bar insets indicate the total distance traveled of the spheres within 15 min after the first contact with the emerging cephalic furrow. **c/d** Additional example of timelapse transmission microscopy of early *Drosophila* embryos during cephalic furrow formation microinjected with IP-PDMS with (c) and without (d) overlays. Magenta dashed lines indicate the gross morphology of the cephalic furrow. Scale bars: 100 µm.

**Fig. S4: Multi-photon 3D laser printed star in bipolar cell layer and contralateral control eyes of the stage 41 medaka embryos shown in Figure 5.**


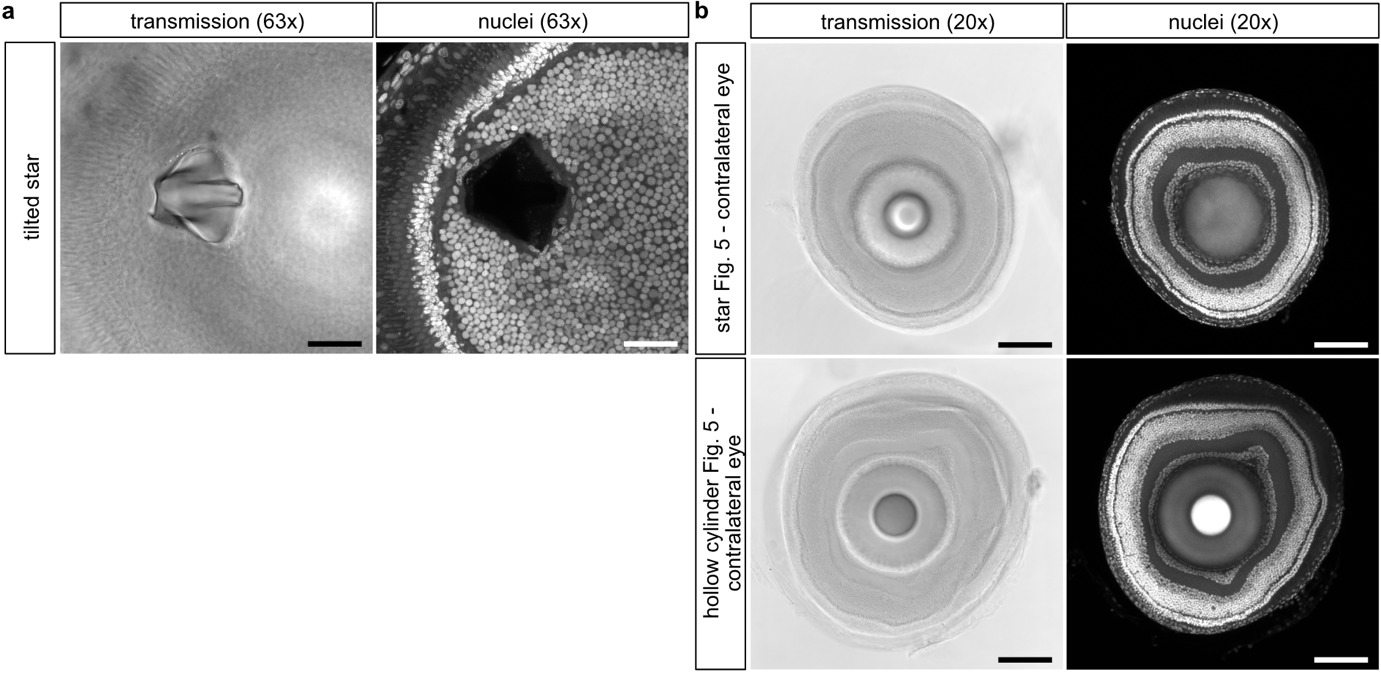


**a** Representative confocal microscopy of chemically fixed and whole-mount nuclear stained (Nuclei; DAPI) medaka embryo eyes at 19 days post fertilization (dpf) after microinjection of IP-PDMS into their optic vesicles and subsequent 3D laser printing at 1dpf. The slightly tilted star measures 60 µm x 60 µm x 20 µm and is integrated into the retinal inner nuclear cell layer (INL). **b** Contralateral, uninjected eyes of the medaka embryos shown in Figure 5b. Scale bars: 100 µm.

**Fig. S5: 3D rendered models of in vivo 3D printed microstructures shown in Fig. 4 and 5.**


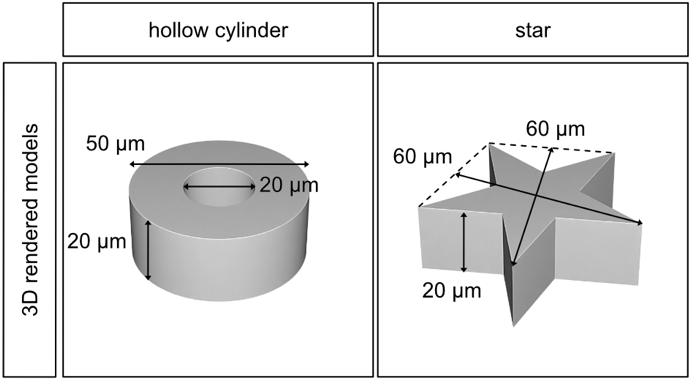


**Movie S1: UV cured spheres at the extreme anterior pole trigger an additional round of anterior nuclear division resulting in a time shift of cellularization onset.**

Representative timelapse transmission microscopy of early *Drosophila* embryos microinjected at the extreme anterior pole with IP-PDMS during cellularization. Microinjected IP-PDMS deposit was cured by UV light exposure. Corresponding timelapse movie to Fig. 3c (right panel). Scale bar: 100 µm.
